# Dr. G. H. Hodge

**Published:** 1874-04

**Authors:** 


Dr. G. D. Hodge, of Holly Springs, Ark, writes: You are altogether right, "Old Spicer," every medical practitioner,

"From the Norther posies,
To the Southern roses,"

*should* take, and read the Southern Medical Recrod."

The Dr. adds he is much pleased with our "New Editorial Staff," and that he will send along a few more subscribers. If *all* our readers will go to work to *simply* add one new name each, the Record will go ahead of any Journal in the country. Wont they try?

